# Accelerated hypofractionated radiotherapy as adjuvant regimen after conserving surgery for early breast cancer: interim report of toxicity after a minimum follow up of 3 years

**DOI:** 10.1186/1756-9966-29-9

**Published:** 2010-01-25

**Authors:** Paola Pinnarò, Antonella Soriani, Valeria Landoni, Carolina Giordano, Maria Papale, Annelisa Marsella, Laura Marucci, Giorgio Arcangeli, Lidia Strigari

**Affiliations:** 1 Radiotherapy Department, Regina Elena National Cancer Institute, Rome, Italy; 2 Laboratory of Medical Physics and Expert Systems, Regina Elena National Cancer Institute, Rome, Italy; 3 Department of Respiratory Physiology, Regina Elena National Cancer Institute, Rome, Italy; 4 Department of Radiology, Regina Elena National Cancer Institute Rome, Italy

## Abstract

**Background:**

Accelerated hypofractionation is an attractive approach for adjuvant whole breast radiotherapy. In this study we evaluated the adverse effects at least 3 years post an accelerated hypofractionated whole breast radiotherapy schedule.

**Methods:**

From October 2004 to March 2006, 39 consecutive patients aged over 18 years with pTis, pT1-2, pN0-1 breast adenocarcinoma who underwent conservative surgery were treated with an adjuvant accelerated hypofractionated radiotherapy schedule consisting of 34 Gy in 10 daily fractions over 2 weeks to the whole breast, followed after 1 week by an electron boost dose of 8 Gy in a single fraction to the tumour bed. Skin and lung radiation toxicity was evaluated daily during therapy, once a week for one month after radiotherapy completion, every 3 months for the first year and from then on every six months. In particular lung toxicity was investigated in terms of CT density evaluation, pulmonary functional tests, and clinical and radiological scoring. Paired t-test, Chi-square test and non-parametric Wilcoxon test were performed.

**Results:**

After a median follow-up of 43 months (range 36-52 months), all the patients are alive and disease-free. None of the patients showed any clinical signs of lung toxicity, no CT-lung toxicity was denoted by radiologist on CT lung images acquired about 1 year post-radiotherapy, no variation of pulmonary density evaluated in terms of normalised Hounsfield numbers was evident. Barely palpable increased density of the treated breast was noted in 9 out of 39 patients (in 2 patients this toxicity was limited to the boost area) and teleangectasia (<1/cm^2^) limited to the boost area was evident in 2 out of 39 patients. The compliance with the treatment was excellent (100%).

**Conclusion:**

The radiotherapy schedule investigated in this study (i.e 34 Gy in 3.4 Gy/fr plus boost dose of 8 Gy in single fraction) is a feasible and safe treatment and does not lead to adjunctive acute and late toxicities. A longer follow up is necessary to confirm these favourable results.

## Background

Breast radiation therapy after conservative surgery is now widely accepted as a standard of care for patients with early breast cancer. Moreover breast conserving therapy has become an accepted treatment option over radical mastectomy for stage I - II breast tumour [1-3].

The conventional radiation course consists of 50 Gy in 25 daily fractions of 2 Gy on the whole breast usually followed by the addition of a boost dose to the tumour bed of 10 to 16 Gy in 5 - 8 daily fractions resulting in overall 6 - 7 week treatment.

However, in certain patient populations like the elderly and those living far from radiation facilities, adjuvant breast radiotherapy appears to be underutilized because of the substantial length of treatment.

Delivering postoperative radiotherapy in a shorter period of time could effectively be much more convenient for these patients. That is, a shorter schedule of radiotherapy, as an accelerated hypofractionated regimen, could indeed improve the use of breast conserving therapy helping to knock down the "logistical barriers"(in terms of age, aged-related morbidity, time, travel difficulties, absence from family and job, cost etc) and consequently providing more women with this option. This accelerated hypofractionated approach is based on the radiobiologic model that a lower total dose delivered in fewer, larger fractions over a shorter period of time is at least as effective as the traditional longer schedule.

The relationship between total dose, fraction size and tissue response is described by the α/β value (expressed in Gy) in Linear Quadratic (LQ) model [4]. Increasing evidence from randomized trials comparing conventional radiotherapy schedules to different hypofractionated ones in whole breast irradiation after conserving surgery show that breast adenocarcinoma may be associated with lower α/β value than previously thought and closer to those of late-reacting healthy tissues [5-9]. The LQ model suggests that, when the α/β ratio for the tumour is similar to that of the surrounding late-responding normal tissue, the hypofractionated regimen may be equally or potentially more effective than the conventional one [10]. On this basis patients at our Institute who refused to spend 6 to 7 weeks in radiotherapy after breast conserving surgery were offered an accelerated hypofractionated radiation therapy schedule consisting of 10 daily fractions of 3.4 Gy to whole breast plus a boost dose of 8 Gy in a single fraction to the tumour bed.

The paper aims to report a preliminary analysis focusing on the early and late skin and lung toxicity after this accelerated hypofractionated regimen. Lung toxicity was investigated in terms of CT density evaluation, pulmonary functional tests, and clinical and radiological scoring.

## Methods

From October 2004 to March 2006, 39 consecutive patients aged over 18 years with pTis, pT1-2, pN0-1 breast adenocarcinoma who underwent conservative surgery and who refused adjuvant conventional radiotherapy regimen (50 Gy in 25 daily fractions to the whole breast followed by 10 - 16 Gy in 5 - 8 daily fractions to the tumour bed) were treated with an adjuvant accelerated hypofractionated radiotherapy schedule. This consisted of 34 Gy in 10 daily fractions over 2 weeks to the whole breast, followed by an electron boost dose of 8 Gy in a single fraction to the tumour bed after 1 week. The protocol has been approved by the local Ethics and Scientific Committee. All patients provided a written informed consent. The median follow-up from the start of radiotherapy was 43 months (range, 36-52 months). The patient and tumour characteristics are listed in Table 1. Data on potential confounding factors such as pulmonary pre-morbidity, smoking habits and adjuvant chemotherapy and/or hormotherapy were also registered for each patient.

**Table 1:** Patient and tumor main characteristics

**Age (years)**	*median (range) *63 (47-81)
**Menopausal status**	
**Pre**	7
**Post**	32
**Smoking habits**	
**Smokers/Ex smokers**	9
**Non smokers**	30
**Histologic type**	
**Invasive ductal**	31
**Invasive lobular**	1
**Mixed ductal/lobular**	1
**Other**	3
**DCIS**	3
**Grading**	
**1**	8
**2**	22
**3**	7
**Not evaluable**	2
**Tumor diameter (mm)**	*median (range) *14 (1-30)
**pT stage**	
**pTis**	3
**pT1 mic**	1
**pT1a**	5
**pT1b**	5
**pT1c**	18
**pT2**	7
**pN stage (not including DCIS)**	
**pN stage**	
**pN0**	31
**pN1 (≤ 3)**	5
**Estrogen receptor status**	
**positive**	37
**negative**	2
**Progesteron receptor status**	
**positive**	34
**negative**	5
**Chemotherapy**	
**Yes**	12
**No**	27
**Ormonotherapy**	
**No**	7
**Tamoxifen**	17
**Anastrozole**	15
**Follow-up (months)**	*median (range) *43(36-52)

Out of 39 patients, 12 (31%) were treated with adjuvant chemotherapy before radiotherapy, either with CMF (cyclophosphamide 600 mg/m^2^, methotrexate 40 mg/m^2^, 5-FU 600 mg/m^2 ^d 1 and d8 q 4 weeks × 6) in 6 patients or FEC (5-FU 600 mg/m^2^, epirubicin 60 mg/m^2^, cyclophosphamide 600 mg/m^2 ^d 1 q 3 weeks × 6) in 2 patients or EC (epirubicin 60 mg/m^2^, cyclophosphamide 600 mg/m^2 ^d1 q 3 weeks × 4) followed by Docetaxel 100 mg/m^2 ^d1 q 3 weeks × 4) in 4 patients. The adjuvant chemotherapy had generally been completed 3 to 4 weeks before starting radiotherapy and before baseline pulmonary function tests.

Adjuvant hormotherapy, with tamoxifen (associated with luteinizing hormone-releasing hormone analogue in 1 patient) or anastrozole, if indicated, was given simultaneously with radiotherapy.

### Radiobiological Considerations

In order to compare the "standard" radiotherapy treatment consisting of 50 Gy in 25 fractions delivered in an overall time of 33 days to our different fractionation schedule of 34 Gy in 10 fractions delivered in an overall time of 12 days, the Normalized Total Dose (NTD) was calculated. The additional dose of 8 Gy in one fraction given to the tumour bed was also considered. Tumour bed irradiation was performed a week after the end of whole breast radiotherapy, so for the "boost" volume the overall treatment time was 19 days and the total dose was 42 Gy.

The normalized total dose (NTD), or the isoeffective dose in 2 Gy fractions, was calculated using the Withers formula [11]:

where D is the total physical dose, d is the dose per fraction, and α/β is the tissue-specific ratio. In this work we assumed an α/β ratio of 3 Gy for late-responding normal tissues (lung), 3.4 Gy for late change in breast appearance, 4.6 Gy [8] and 10 Gy for tumor control and 10 Gy for skin (considering early reaction). To take into account the different overall treatment time the NTD was corrected in NTD_T _according with the formula [12]:

where "T" is the overall treatment time (in days) for the schedule under consideration that delivered a normalized total dose NTD; "t" is the overall treatment time of a conventionally fractionated scheme (2 Gy/fraction, five fractions/week) that would deliver a radiation dose equal to "NTD_t_".

"t" was calculated as follows: t = ((NTD/2) × (7/5), subtracting 2 days for the weekend if necessary.

The difference (t -T) has positive values for treatment abbreviation and indicates the days of acceleration. D_prolif _is the dose recovered per day due to proliferation, to compensate for rapid cell repopulation. For cancer a D_prolif _value of 0.7 Gy/d was considered, as a mean value in the range 0.5 - 0.9 estimated for most tumours from a review of studies in literature [12,13]. For normal tissues, a D_prolif _value of 0.2 Gy/d was adopted as reported by other authors [14,15].

In Table 2 the results of the radiobiological calculation are summarised. Thus, correcting differences in overall treatment time, our schedule of 34 Gy in 10 fractions plus a boost of 8 Gy in one fraction delivered within 19 days is biologically equivalent to 59-70 Gy in 2 Gy/fr, considering the tumour bed volume, according to the α/β values of 10 and 4.6 Gy, respectively.

**Table 2:** Radiobiological equivalence of schedule used in this study.

Treatment		Breast	Tumor bed
Schedule	d(Gy) × (n. fr)	3.4 × 10	3.4 × 10 plus 8 × 1
	Total physical dose (Gy)	34	42
	Treatment time (days)	12	19

**Normal tissue - late effect**			

Lung (α/β = 3 Gy)	NTD_2 _(Gy)	43.5	61.1
	Acceleration (days) * D_prolif_	18 * 0.2	24 * 0.2
	NTD_T _(Gy)	47.1	65.9
Normal breast (α/β = 3.4 Gy)	NTD_2 _(Gy)	42.8	59.7
	Acceleration (days) * D_prolif_	17 * 0.2	21 * 0.2
	NTD_T _(Gy)	46.2	63.9

**Tumor**			

Breast Tumor (α/β = 4.6 Gy)	NTD_2 _(Gy)	41.2	56.5
	Acceleration (days) * D_prolif_	17 * 0.7	19 * 0.7
	NTD_T _(Gy)	53.1	69.8
Cancer cells (α/β = 10 Gy)	NTD_2 _(Gy)	38	50
	Acceleration (days) * D_prolif_	13 * 0.7	14 * 0.7
	NTD_T _(Gy)	47.1	59.8

**Abbreviations: **NTD_2 _is the normalized total dose at 2 Gy fraction, NTD_T _is the normalized total dose at 2 Gy fraction corrected for time acceleration (see text). Acceleration indicates the difference (t -T) respect to the conventional treatment. D_prolif _is the dose recovered per day due to proliferation, to compensate for rapid cell repopulation.

### Radiotherapy Treatment

Patients were treated in a breast board in the supine position with both arms extended overhead and supported by a dedicated arm rest. 3D Treatment plans (Eclipse Treatment Planning System- Varian CA) were based on CT images acquired by a dedicated radiotherapy AQ Sim CT scan (Philips Medical systems, Netherlands) with a 5 mm spacing from the apex of the lungs to the diaphragm, including the whole lung and breast.

The Clinical Target Volume (CTV) consisted of the whole breast parenchyma. The Planning Target Volume (PTV) was obtained by adding a 1 cm margin to the CTV except in the direction of the skin's surface.

Organs at risk (OARs) such as omolateral lung - from the apex to the base - and the heart in the left-side breast cancer were also outlined in every slice.

3D conformal radiotherapy was delivered by two opposed 6 MV photon beams (Varian LINAC 2100 endowed with a Millenium multileaf collimator). Wedge compensation was used to ensure a uniform dose distribution to the target volume of -5% and +7% [16]. The total dose was 34 Gy delivered in 10 daily fractions, 3.4 Gy per day, 5 days a week; the dose was normalized at the ICRU (International Commission on Radiation Units and Measurements) reference point [16].

Portal images were taken to check positioning just before the first session and then every two sessions.

The boost dose of 8 Gy (prescribed to the 90% reference isodose) was administered in a single fraction by a 6 to 12 MeV electron field according to the location of the tumour bed defined by metallic clips purposefully positioned at the time of the surgery and/or by computer tomography analysis. Dose on the lungs (considering only the homolateral) was kept below the limit of 15.6 Gy to no more than 12.5% of the volume, 10.1 Gy to no more than 14.5% and 7.8 Gy to no more than 16% (Table 3, i.e equivalent to V20 Gy<12.5%, V13<14.5% and V10<16% respectively at 2 Gy/fr regime considering an α/β value for the lung equal to 3 Gy [17,18]).

**Table 3:** Volume and dosimetric parameters related to lung

	Minimum	Average ± sd	Maximum
**Lung Volume (cm^3^)**	807	1403 ± 305	2050
**Mean Lung Dose (Gy)**	0.76	1.69 ± 0.7	4.44
**V_7.8 _Gy (%)**	1.1	4.5 ± 2.3	13.0
**V_10.1_Gy (%)**	0.9	4.1 ± 2.1	12.2
**V_15.6_Gy (%)**	0.6	3.4 ± 1.9	10.9
**Maximum lung distance (mm)**	2	14 ± 4	23

**Abbreviations: **sd = standard deviation, V_x _= the % of lung volume receiving at least the dose X in Gy.

Dose-volume histograms (DVHs) analysis were calculated and registered for all OARs.

### Pulmonary function tests (PFTs)

Pulmonary function tests were performed before the beginning of radiotherapy and then after 6, 12 and 24 months from the end of radiotherapy.

Forced Vital Capacity (FVC), Forced Expiratory Volume in 1 s (FEV1) and Carbon Monoxide Diffusing Capacity (DLCO) acquired with the single breath technique have been measured with a Quark PFT Cosmed spirometer. Measurements are expressed as absolute and relative values (normalised for weight, height, sex and age of patients in agreement with ERS 93 parameters) [19].

Hemogas analysis was performed with blood sampling from the radial arteria or omeral arteria and analysed with the ABL 520 blood gas analyzer system.

The PaO_2,ST _(i.e. standard) was standardized to a PaCO_2 _of 40 mmHg from the PaO_2 _and PaCO_2 _values and corrected for the effect of hyperventilation [20].

The evaluation of pulmonary functionary was performed on 38 patients (one patient refused post-radiotherapy PFTs and one-year post-radiotherapy CT). Nine patients were, or had been in the past, smokers.

### Toxicity

Radiation toxicity was evaluated daily during therapy, once a week for one month after radiotherapy completion, every 3 months for the first year and from then on every six months.

The National Cancer Institute Common Toxicity Criteria, version 2, was used to assess the acute toxicity [21].

The SOMA/LENT scoring system was used for the assessment of late sequelae [22].

The National Cancer Institute Common Toxicity Criteria, version 4, was used to assess the lung toxicity based on pulmonary function tests [23].

### CT scan evaluation

In order to evaluate density of omolateral lung, a chest CT scan (with the patient in the same treatment position) was planned about one year post-radiotherapy. Out of 39 patients, 38 underwent chest CT scans before (1 patient refused one-year post-radiotherapy CT and post-radiotherapy PFTs).

A radiologist with specific experience (blinded to the side of irradiation) was asked to assess differences between the two lungs and to score CT- lung alteration according to Nishioka et al. [24] scoring system, summarized as follows. Grade 0: no significant changes in the radiation fields; Grade 1: only pleural thickening is seen in the radiation fields; Grade 2 pulmonary changes (plaque-like or heterogeneous density) are seen in less than 50% area of the radiation fields; Grade 3: pulmonary changes are seen in more than 50% area of the radiation fields.

We also evaluated the radiation induced pulmonary density changes by a modified Wennemberg et al. [25] CT-based method. The CT scan performed before radiotherapy for treatment planning and the one-year follow-up CT scan were considered. On both sets two levels were examined: CT slices corresponding to the isocenter and the boost area. For lung evaluation, regions of interest of about 1 cm diameter immediately below the thoracic wall were drawn in the irradiated and non irradiated lung and in the pre-radiotherapy and post-radiotherapy (1 year after) CT scan. The mean density and the standard deviation within the area of interest were calculated by TPS tools. Density was evaluated in Hounsfield Units (HU) representing the mean attenuation of the tissue examined, in a scale where -1000 and 0 are the air and the water density values, respectively. To correct lung density differences due to breathing, the contra-lateral mean lung density value was subtracted from that of the omolateral lung (normalized HU). Differences between HU values before and after radiotherapy were assessed for each patient.

### Statistical analysis

A t test and Chi-square test were performed to investigate whether there was any correlation between the measurements of pulmonary fibrosis through the method of Hounsfield numbers, chemotherapy (CT), smoking history (current and ex smokers vs. Non-smokers), age and dosimetric parameters. The dosimetric parameters investigated were MLD (the mean lung dose expressed in Gy), V15.6 Gy, V7.8 Gy, V3.6 Gy (the % of lung volume receiving at least 15.6 Gy, 7.8 Gy and 3.6 Gy, respectively).

The non-parametric Wilcoxon test for paired samples was performed between data of FPT parameters recorded before and after treatment. A p-value < 0.05 was considered statistically significant.

## Results

After a median follow-up of 43 months (range, 36-52 months), all the patients are alive and disease-free. There were no major nor minor treatment deviations resulting in 100% compliance with the treatment.

**Figure 1:**
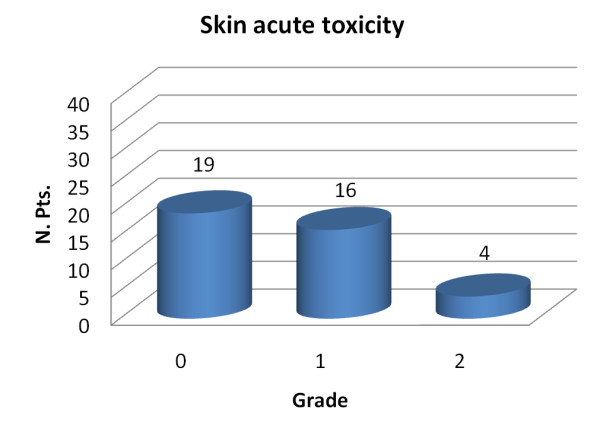
**Skin acute toxicity based on ctc v.2 criteria versus toxicity grade observed for the 39 patients**.

Acute skin toxicity against the grade evaluated according to the CTC v.2 criteria is shown in Figure 1.

Of the 39 patients, 19 (49%) had no acute skin toxicity at all, 16 (41.0%) had Grade 1, consisting in all cases in faint erythema, and 4 patients (10%) presented Grade 2 toxicity consisting in moderate erythema. The peak incidence of Grade 2 acute skin toxicity occurred at 1 week after the treatment ending with two patients having reactions confined to the boost area. No patient suffered Grade 3 or more acute skin toxicity. Neither was there any correlation found between acute skin toxicity and breast volume nor previous adjuvant chemotherapy (with or without antracyclines).

**Figure 2:**
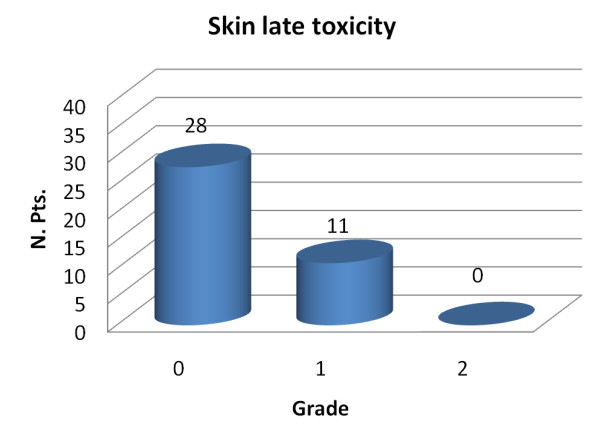
**Skin late toxicity based on ctc v.2 criteria versus toxicity grade for the 39 patients**.

Figure 2 summarized late breast toxicity according to the SOMA/LENT scoring system.

At the time of analysis with a minimum follow- up of 36 months, Grade 1 late breast toxicity was present in 11 patients (28%) and consisted of barely palpable increased density in nine patients (in 2 patients this toxicity was limited to the boost area) and teleangectasia (<1/cm^2^) limited to the boost area in 2 patients. No toxicity grade 2 or more was observed. Also in this case no correlation was found with breast volume and with previous adjuvant chemotherapy.

**Figure 3:**
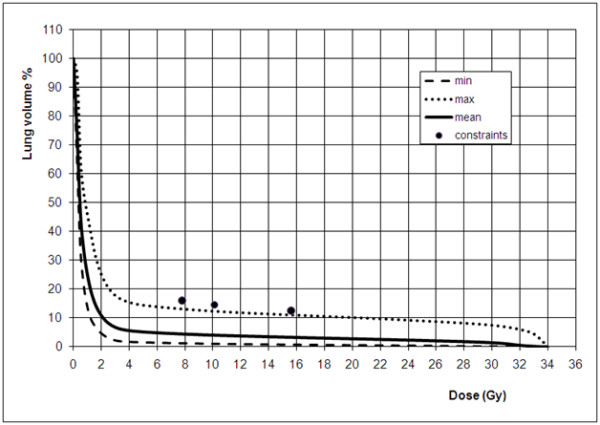
**Minimum (broken line), mean (solid line), maximum (dotted line) cumulative lung dose volume histograms for hypofractionated breast radiotherapy**. Filled circles indicate dose volume constraints used for planning, equivalent to V20 Gy<12.5%, V13<14.5% and V10<16% respectively at 2 Gy/fr regime considering an α/β value for the lung equal to 3 Gy.

In Figure 3 the mean dose volume histogram for the lung is shown together with the less and most favorable histograms, dose volume constraints in terms of 2 Gy per fraction equivalence are always respected.

None of the 39 patients presented symptoms of radiation pneumonitis or any other respiratory symptoms (coughing and/or dyspnea with or without fever) or problems judged by the clinician to be caused by radiotherapy.

No CT-lung toxicity according to Nishioka et al.[24] scoring system was denoted by radiologist on CT lung images acquired about 1 year post-radiotherapy.

A t-test was performed to investigate the correlation between the variation of pulmonary density evaluated in terms of normalised Hounsfield numbers and age, hormonal treatment and dosimetric parameters (p > 0.05, data not shown). No significant correlation was found with chemotherapy (p > 0.05) as it can be seen from the results reported in Table 4.

**Table 4:** Hounsfield values in ROIs delineated on CT images before and post-RT.

	*chemotherapy*	*no chemotherapy*	*p-value (t-test)*
	(average ± sd)	(average ± sd)	
Isoplan pre-RT	-815 ± 32	-817 ± 32	0.419
isoplan post-RT	-813 ± 43	-818 ± 29	0.325
boost post-RT	-789 ± 49	-810 ± 47	0.118

The potential impact of the treatment on breathing was investigated (Table 5).

**Table 5:** DLCO and FEV1% measured before and at 2 year post-radiotherapy against chemotherapy, TAM and smoking habits.

*Adverse Event*	*group*	*Percentage of ≥G1 grade*	*p (§)*	*Percentage of ≥G2 grade*	***p (§***)
**DLCO measured before radiotherapy respect to predicted value for each patient**					
	Chemotherapy vs no chemotherapy	78% vs. 22%	***0.006***	38% vs 6%	***0.036***
	TAM vs no TAM	43% vs. 44%	0.755	14% vs 17%	0.972
	Smoking vs no smoking	67% vs. 31%	0.111	44% vs 19%	0.299
**DLCO measured at 2 year post-radiotherapy respect to predicted value for each patient**					
	Chemotherapy vs no chemotherapy	67% vs. 41%	0.251	45% vs 19%	0.258
	TAM vs no TAM	44% vs. 52%	0.848	25% vs 29%	0.993
	Smoking vs no smoking	54% vs. 46%	0.930	31% vs 17%	0.538
**FEV1% measured before radiotherapy respect to predicted value for each patient**					
	Chemotherapy vs no chemotherapy	40% vs. 42%	0.765	0% vs. 0%	-
	TAM vs no TAM	36% vs. 43%	0.996	0% vs. 0%	-
	Smoking vs no smoking	40% vs. 41%	0.882	0% vs. 0%	-
**FEV1% measured at 2y-post-radiotherapy respect to predicted value for each patient**					
	Chemotherapy vs no chemotherapy	44% vs. 50%	0.890	0% vs. 4%	0.673
	TAM vs no TAM	44% vs. 56%	0.464	0% vs 6%	0.853
	Smoking vs no smoking	62% vs. 5%	***<0.001***	0% vs 5%	0.931

(§) p-value chi-square test

In particular a ≥G1 toxicity based on DLCO was observed in 78% and 22% of patients who did/did not receive adjuvant chemotherapy before radiotherapy, respectively (p = 0.006, Table 5). The ≥G2 toxicity based on DLCO was observed in 38% and 6% of patients who did/did not receive adjuvant chemotherapy before radiotherapy, respectively (p = 0. 034, Table 5). These differences were lost both for ≥G1 than for ≥G2 at 2-year post-radiotherapy, indicating a recovery over time of the capacity of diffusivity.

No correlation was observed in toxicity (grade ≥G2 and ≥G1) based on DLCO with TAM while a correlation was observed with smoking habits before radiotherapy.

FEV1%, expressed as a percentage in comparison to the predicted value for each patient, before and at 2 years post-radiotherapy was not statistically different in patients who did or did not receive chemotherapy. No correlation was observed with TAM while a significant correlation was found with smoking habits for ≥G1 at 2-years post-radiotherapy (Table 5). In particular a ≥G1 toxicity based on FEV1% was observed in 62% and 5% of smokers/non smokers, respectively (p < 0.001).

## Discussion

Breast radiation therapy after conservative surgery is now widely accepted as a standard of care for patients with early breast cancer. Moreover breast conserving therapy has become an accepted treatment option over radical mastectomy for stage I - II breast tumour.

However, in some patients, such as the elderly and those living faraway from radiation facilities, adjuvant breast radiotherapy appears to be underutilized because of the substantial length of the standard radiation course. This usually consists of 50 Gy in 25 daily fractions of 2 Gy to the whole breast usually followed by the addition of a boost dose to the tumour bed of 10-16 Gy in 5 - 8 daily fractions, resulting in an overall treatment time of 6 - 7 weeks. Delivering postoperative radiotherapy in a shorter time could effectively be much more convenient for these patients knocking down the "logistical barriers" to the adjuvant breast radiotherapy. Several clinical randomized trials have shown that hypofractionated adjuvant radiotherapy in breast cancer offers similar rates of tumour control and normal tissue damage as the standard schedule [7-9].

In our Institute patients refusing a 42-49 day lasting treatment were offered an accelerated hypofractionated schedule requiring 19 days. Despite this "aggressiveness" the radiotherapy schedule investigated in this study (i.e 34 Gy in 3.4 Gy/fr plus boost dose of 8 Gy in single fraction) was well tolerated and compliant. It is worthwhile to note that the early and late radiation toxicity appeared remarkably low and comparable to standard regime.

In particular, acute skin toxicity of Grade 0, 1, and 2 was experienced by 49%, 41.0% and 10% of patients respectively; no patient experienced Grade 3 or more. This toxicity was much lower than expected from standard radiotherapy [26].

G1 late skin toxicity was observed in 11 out of 39 patients with no G2 or more.

No correlation between chemotherapy and skin toxicity was found. However, due to the low number of patients receiving chemotherapy (12/39) and the different schedules of chemotherapy (CMF or FEC or EC followed by Docetaxel) used, further patients are needed to confirm this finding.

No patient referred symptoms of radiation pneumonitis or other respiratory symptoms or problems clinically related to radiotherapy. No CT-lung toxicity was denoted by the radiologist on CT-scans acquired at 1 year post-radiotherapy. These results are comparable with those reported in other studies where early and late lung toxicity after standard or hypofractionated radiotherapy to the breast only was studied [15,27,28].

Bentzen et al. reported enhanced RT-induced pulmonary fibrosis in patients treated with concomitant tamoxifen [29]. This effect was not observed in our cohort of patients.

In accordance with Wennemberg et al. [25] no correlation was found between HU and either chemotherapy or TAM.

Nevertherless the very low incidence of lung toxicity was certainly also related, in our series, to the very low values of doses administered to the lung volume as shown from the calculated dose volume histograms.

Statistically significant changes in toxicity ≥G2 and ≥G1 based on DLCO (p = 0.006 and p = 0.034, respectively) were detected when comparing data of patients who did receive chemo-therapy and those who did not, but no adjunctive effects were seen due to radiotherapy. These findings are in accordance with the low observed mean DLCO caused by the adjuvant chemotherapy [27,30]. This confirms that DLCO is a more sensitive variable of functional pulmonary changes due to drug-induced toxicity.

These differences were lost at 2 years post-radiotherapy indicating recovery over time and no additional influence of hypo-fractionated radiotherapy schedule. These results confirm the literature [27], indicating a trend towards normalization at 5 months after radiotherapy.

FEV1% showed a significant correlation with smoking habits for ≥G1 toxicity at 2-years post-radiotherapy.

Our findings support the hypothesis that this new hypofractionated schedule neither implies a detriment of functional breathing nor hinders the recovery over time.

## Conclusion

The radiotherapy schedule investigated in this study (i.e 34 Gy in 3.4 Gy/fr plus boost dose of 8 Gy in single fraction) is a feasible and safe treatment and does not lead to adjunctive acute and late toxicities. A longer follow up is expected to confirm these favourable results. Still, randomized prospective studies, designed to validate accelerated hypofractionated schedules, should be encouraged.

## Competing interests

The authors declare that they have no competing interests.

## Authors' contributions

PP and GA made conception, designed and coordinated the study, collected samples, analyzed data, carried out data interpretation, and drafted the manuscript. CG and LM performed the revaluation of clinical toxicity, collected samples and evaluated the results. MP performed the pulmonary functional test and evaluated the results. AM performed the revaluation of radiological toxicity and evaluated the results. VL, AS and LS participated in the conception, analyzed data, carried out data interpretation, design of study and in drafting of manuscript. All authors read and approved the final manuscript.
